# Comparison of detection frequencies between ultrasonography and abdominal radiography for abnormal signs in necrotizing enterocolitis: a systematic review and meta-analysis

**DOI:** 10.3389/fped.2026.1889902

**Published:** 2026-07-29

**Authors:** Jinglan Huang, Qian Gao, Ziqing Xu, Xin Li

**Affiliations:** 1 Department of Pediatrics, West China Second University Hospital, Sichuan University, Chengdu, China; 2 Key Laboratory of Birth Defects and Related Diseases of Women and Children of the Ministry of Education, Sichuan University, Chengdu, Sichuan, China

**Keywords:** abdominal radiography, abnormalities, necrotizing enterocolitis, neonatal, signs, ultrasonography

## Abstract

**Objective:**

To synthesize the existing evidence regarding the differences between abdominal ultrasound (AUS) and conventional radiography in detecting necrotizing enterocolitis (NEC) features in neonates.

**Methods:**

A six-database literature search was conducted to identify relevant English-language studies published prior to April 1, 2026 comparing the detection frequencies of AUS and abdominal x-ray (AXR) for imaging signs of neonatal NEC.

**Results:**

Twenty studies were identified that systematically compared the detection frequencies of AUS vs. AXR for detecting specific NEC features (*n* = 1,793 neonates with confirmed or suspected NEC). AUS detected portal venous gas more frequently than AXR [odds ratio (OR): 2.32; 95% confidence interval (CI) [1.35–3.99]] and exhibits a higher, albeit not significant, detection frequency for pneumatosis intestinalis [OR: 1.21; 95% CI (0.82–1.80)]. AXR detected pneumoperitoneum [OR: 0.62; 95% CI (0.31–1.26)] and intestinal dilatation [OR: 0.51; 95% CI (0.19–1.40)] more frequently than AUS, though neither frequency was statistically significant.

**Conclusions:**

AUS and AXR show different patterns of detection of abnormal signs in NEC, with the former detecting portal gas and pneumatosis intestinalis more frequently than the latter. Combining the two modalities in clinical practice may facilitate earlier recognition and assessment of disease progression in neonatal NEC cases.

## 1 Introduction

In recent years, advances in medical technology have led to significant improvements in the survival rates of preterm and low-birth-weight (LBW; <2,500 g) infants (1). Global data from 10 countries suggest that the mean survival rate among infants with very LBW (<1,500 g) is approximately 87.0%; however, recent data from China indicate that survival rates can reach as high as 95.4% among infants born before 32 weeks of gestation if comprehensive treatment regimens are implemented (2, 3). Despite the improved survival rates that such interventions can provide, preterm and very-LBW infants may still experience various complications, including necrotizing enterocolitis (NEC) (1, 4). In severe cases, this severe inflammatory condition can progress rapidly to require surgical intervention, and it is a significant contributor to mortality among preterm and LBW infants (5). Therefore, early recognition and aggressive management of NEC are important for improving survival rates in these patients.

Currently, the diagnosis of NEC is primarily based on clinical manifestations and imaging findings from abdominal x-ray (AXR). In infants presenting with symptoms such as vomiting, abdominal distension, or bloody stool, AXR may help identify signs of intestinal dilatation, pneumatosis intestinalis, or pneumoperitoneum, which can be useful for guiding clinical decision-making regarding treatment strategies (6). However, owing to technological advances in ultrasonography, studies have shown that abdominal ultrasound (AUS) could help facilitate the early assessment of intestinal perfusion, bowel wall thickness, and motility in pediatric patients (7–9). These capabilities suggest that AUS may identify certain NEC-related findings more readily than AXR, potentially guiding the timing of surgery and allowing clinical interventions to be initiated sooner.

The primary objective of this study was to synthesize the current evidence by systematically comparing the detection capabilities of AUS and AXR in NEC. The findings could help facilitate the identification of an optimized imaging approach for recognizing NEC features and risk stratification in neonates with this affliction.

## 2 Materials and methods

### 2.1 Search strategy

This systematic review adhered to the Preferred Reporting Items for Systematic Reviews and Meta-Analyses (PRISMA) guidelines (10). The study protocol was prospectively registered in the International Prospective Register of Systematic Reviews (PROSPERO) database (registration number: CRD420251147314). We searched for the relevant literature published in six English databases (EBSCO, Embase, Web of Science, Cochrane Library, PubMed and Google Scholar databases) before April 1, 2026, comparing the findings of AUS and AXR in diagnosing NEC in newborns. We used the keywords (“Necrotizing Enterocolitis” OR “NEC” OR “Enterocolitis, Necrotizing”) AND (“Ultrasound” OR “Ultrasonograph” OR “Sonograph” OR “Doppler”) AND (“radiography” OR “x-ray”). Additionally, the reference lists of included studies were reviewed.

### 2.2 Study selection

Two researchers independently screened the titles and abstracts of potentially eligible studies prior to conducting full-text assessments. Any discrepancies were resolved through discussion or consultation with a third reviewer. Studies were included based on the following eligibility criteria: (1) they involved neonates with diagnosed or suspected NEC; (2) they compared AUS with AXR; (3) they were randomized controlled trials (RCTs), case-control studies, or cohort studies; and (4) they were published in English. Review articles, systematic reviews, case reports, editorials, animal studies, duplicate publications, and studies with incomplete data were excluded.

### 2.3 Data extraction

Two researchers extracted data from the eligible studies, including the name of the first author, publication year, study location, study design, sample size, patients' GA, and NEC definition used. Disagreements were adjudicated either by establishing a consensus between the two researchers or, when that was not possible, through consultation with a third investigator.

### 2.4 Assessment of publication bias

To evaluate potential publication bias, funnel plots were visually inspected for evidence of asymmetry, and Egger's test was conducted as an objective means of verification. The threshold of statistical significance for the presence of publication bias was *p* < 0.05.

### 2.5 Quality evaluation

The methodological quality of the observational studies included in the analysis was evaluated using the Newcastle-Ottawa Scale (NOS) (11). Two researchers independently scored each study based on selection, comparability, and outcome domains, and each study was categorized as either high quality (7–9 points), moderate quality (5–6 points), or low quality (≤4 points). RCTs were evaluated using the Cochrane Risk of Bias tool (RoB 1) and categorized as having low, unclear, or high risk of bias. Studies with low risk of bias in all domains were considered high quality. Any disagreements in scoring were adjudicated by a third investigator.

### 2.6 Sensitivity analysis

Two approaches were adopted for the sensitivity analyses, including the leave-one-out method and the exclusion of studies with an NOS score ≤ 7. To account for within-subject correlation in paired-design studies, we performed sensitivity analyses using variance inflation with intraclass correlation coefficients (ICCs) of 0.3 and 0.5, restricted to studies that applied both modalities to the same cohort.

### 2.7 Subgroup analyses

To explore sources of heterogeneity, we performed subgroup analyses according to (1) gestational age (neonates vs. preterm infant vs. mean <32 weeks), (2) NEC definition (Bell's or modified Bell's criteria vs. definition by Walsh and Kliegman vs. no clear definition), and (3) surgical verification status (surgical verification vs. clinical diagnosis). Subgroup analyses were conducted for outcomes with at least two studies per subgroup. The <32 weeks subgroup was nested within the preterm infant category and therefore was not treated as a separate stratum in the subgroup interaction analyses.

### 2.8 GRADE assessment

The Grading of Recommendations Assessment, Development, and Evaluation (GRADE) approach was used to assess the quality of evidence for each outcome (12). For observational studies, the initial certainty of evidence was rated as low by default. It could be downgraded for study limitations, inconsistency, indirectness, imprecision, or publication bias, and upgraded if the study reported a large magnitude of effect (e.g., relative risk >2 or <0.5) or exhibited a dose–response gradient with a statistically significant test for trend.

### 2.9 Statistical analysis

All statistical analyses were performed using Review Manager 5.3 (The Cochrane Collaboration, London, UK) and Stata 12.0 (StataCorp, College Station, TX, USA). The relative detection frequencies of AUS and AXR are expressed as odds ratios (ORs) with 95% confidence intervals (CIs), representing the odds of sign detection by AUS relative to AXR. Considering the expected heterogeneity, a random effects model (using the generic inverse variance method) was applied. Heterogeneity was assessed using the Q statistic (with *p* < 0.10 considered statistically significant) and the I² statistic, where values of 25%, 50%, and 75% were interpreted as being indicative of low, moderate, and high heterogeneity, respectively. Subgroup analyses were performed to explore potential sources of heterogeneity. All subgroup analyses were exploratory and were not adjusted for multiple comparisons. For outcomes in which zero cases were detected in the AXR group in more than half of the included studies, it was infeasible to conduct a valid meta-analytic pooling; consequently, these outcomes could only be summarized descriptively.

## 3 Results

### 3.1 Study characteristics

**Figure 1:**
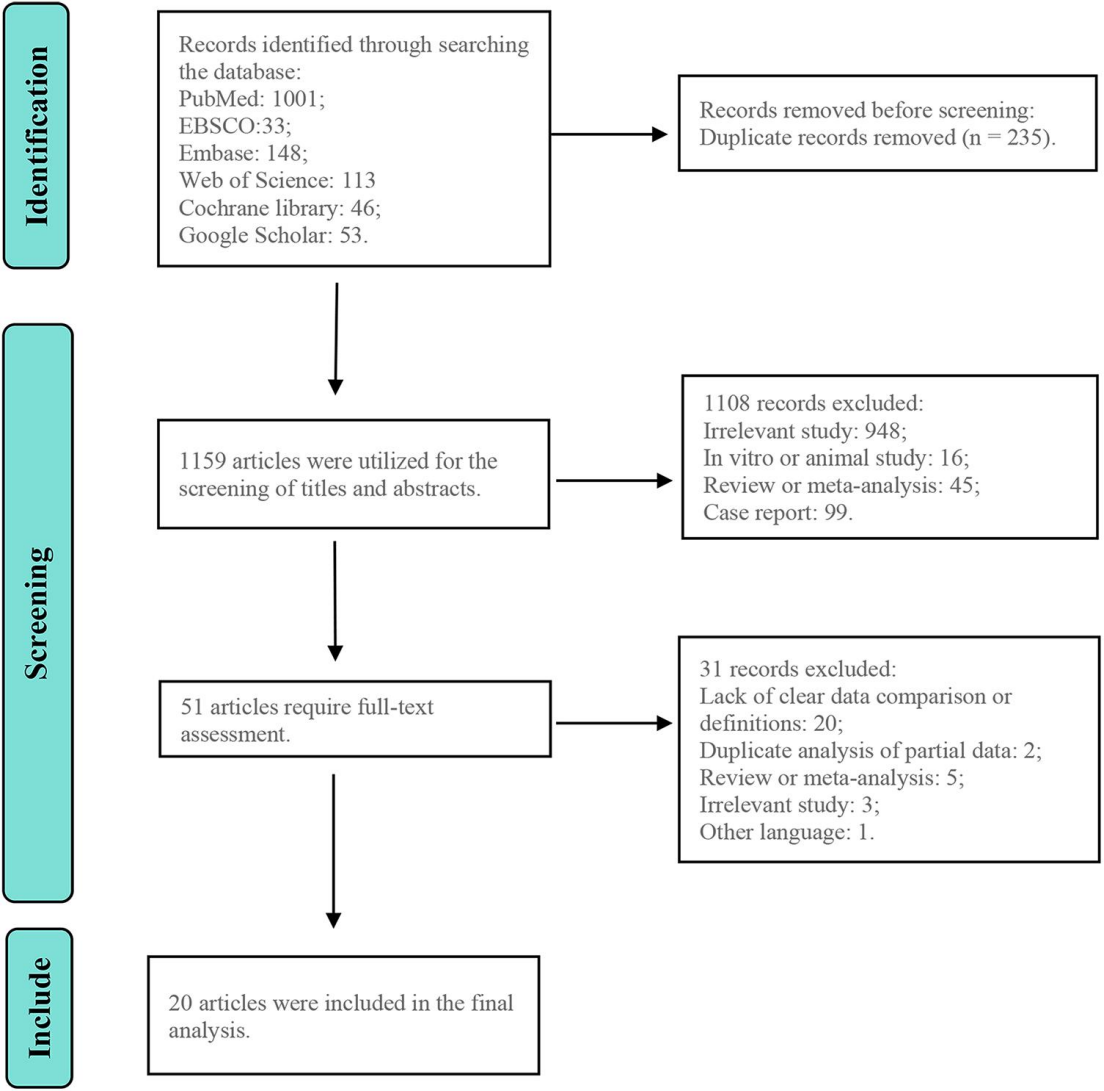
The flow diagram of studies selection.

A total of 1,394 publications were identified from the date of inception of the databases to April 1, 2026. After the removal of 235 duplicate records, a further 1,108 studies were excluded during the title and abstract screening process. The remaining 51 articles underwent full-text review, 31 of which were excluded based on the following criteria: having insufficient data for comparison; having overlapping patient populations (partial duplication); having a non-primary study design (e.g., reviews); reporting irrelevant outcomes; and being published in a non-English language. Ultimately, 20 studies were included in the meta-analysis, including 19 observational studies (7–9, 13–28) and one RCT (29), comprising a total of 1,793 neonates (Figure 1). The characteristics of the included studies are summarized in Table 1. Among the 19 observational studies, NOS scores ranged from 7 to 9 (median: 8). Four studies scored 7 points (borderline high quality), and the remaining 15 scored 8 or 9 points. Thus, according to our three-tier classification, all 19 observational studies met the threshold for high quality (≥7 points). The RCT was assessed using the Cochrane Risk of Bias tool and was rated as having a low risk of bias. The item-level NOS scores for each observational study are presented in detail in Table 2 and Supplementary Figure S1. Of the 20 included studies, 17 applied both AUS and AXR to the same patient cohort (paired design), whereas three applied each modality to a different patient group (unpaired design). A detailed classification is provided in Supplementary Table S1.

**Table 1:** The characteristics of studies included in the meta-analysis.

Study (author, year)	Country	Study design	Number	Patients GA^b^	NEC diagnosis	Definition of NEC^a^
Sur (7)	UK	Cohort Study	28	mean GA = 28.4 weeks	clinical + radiographic	Bell's criteria
Vallant (8)	UK	Cohort Study	39	GA <24 weeks	clinical + radiographic + surgical	No clear definition
Oulego-Erroz I, 2022	Spain	Case-control Study	84	GA < 36 weeks	clinical + radiographic	VON case definition or the “two of three rules” case definition
Mathews (22)	India	Cohort Study	200	neonates	clinical + radiographic + surgical	Modified Bell's criteria
Kim (20)	Korea	Case-control Study	57	mean GA = 24.6 weeks	clinical + radiographic + surgical	No clear definition
Yu (28)	China	Cohort Study	184	neonates	clinical + radiographic + surgical	Revised Bell's clinical diagnostic criteria
Lazow (21)	USA	Case-control Study	86	neonates	clinical + radiographic + surgical	No clear definition
Cuna (29)	USA	Randomized clinical trial	16	GA ≤ 32 weeks	clinical + radiographic	No clear definition
Gao (17)	China	Case-control Study	213	GA ≤ 36 weeks	clinical + radiographic	Bell's NEC classification standard
Tracy (26)	USA	Cohort Study	162	GA < 32 weeks	clinical + radiographic + surgical	No clear definition
Chen (14)	China	Case-control Study	86	preterm infant	clinical + radiographic	NEC definition by Walsh and Kliegman
Aliev (13)	Uzbekistan	Cohort Study	51	preterm infant	clinical + radiographic + surgical	Bell's NEC classification standard
Wang (27)	China	Cohort Study	144	neonates	clinical + radiographic	Modified Bell's criteria
Staryszak (25)	Poland	Cohort Study	9	neonates	clinical + radiographic	Bell's criteria
Kamali (19)	Iran	Case-control Study	67	GA < 37 weeks	clinical + radiographic	Modified Bell's criteria
Garbi-Goutel (18)	France	Case-control Study	95	GA < 33 weeks	clinical + radiographic	Bell's criteria
Silva (24)	Canada	Cohort Study	75	neonates	clinical + radiographic	Bell's criteria
Muchantef (23)	USA	Case-control Study	44	neonates	clinical + radiographic + surgical	No clear definition
Dilli (15)	Turkey	Case-control Study	93	preterm infant	clinical + radiographic + surgical	NEC definition by Walsh and Kliegman
Faingold (16)	Canada	Case-control Study	60	preterm infant	clinical + radiographic	Modified Bell staging criteria

a NEC, Necrotizing enterocolitis.

b GA, gestational age.

**Table 2:** Quality assessment of the included observational studies.

Study (first author and year)	Study design	Selection			Comparability			Outcome/Exposure			Total score
Sur (7)	Cohort Study	1	1	1	1	1	1	1	1	1	9
Vallant (8)	Cohort Study	1	1	1	1	1	1	1	1	1	9
Oulego-Erroz I, 2022	Case-control Study	1	1	1	1	1	1	1	1	1	9
Mathews (22)	Cohort Study	1	1	1	1	1	1	1	1	1	9
Kim (20)	Case-control Study	1	1	1	1	1	1	1	1	1	9
Yu (28)	Cohort Study	1	1	1	1	0	0	1	1	1	7
Lazow (21)	Case-control Study	1	1	1	1	0	0	1	1	1	7
Gao, (17)	Case-control Study	1	1	1	1	1	0	1	1	1	8
Tracy (26)	Cohort Study	1	1	1	1	1	1	1	1	1	9
Chen (14)	Case-control Study	1	1	1	1	1	1	1	1	1	9
Aliev (13)	Cohort Study	1	1	1	1	1	1	1	1	1	9
Wang (27)	Cohort Study	1	1	1	1	1	1	1	1	1	9
Staryszak (25)	Cohort Study	1	1	1	1	1	1	1	1	1	9
Kamali (19)	Case-control Study	1	1	1	1	1	1	1	1	1	9
Garbi-Goutel (18)	Case-control Study	1	1	1	1	0	0	1	1	1	7
Silva (24)	Cohort Study	1	1	1	1	0	0	1	1	1	7
Muchantef (23)	Case-control Study	1	1	1	1	1	1	1	1	1	9
Dilli (15)	Case-control Study	1	1	1	1	1	1	1	1	1	9
Faingold (16)	Case-control Study	1	1	1	1	0	1	1	1	1	8

### 3.2 Comparison of the ability of AUS and AXR to detect NEC features

**Figure 2:**
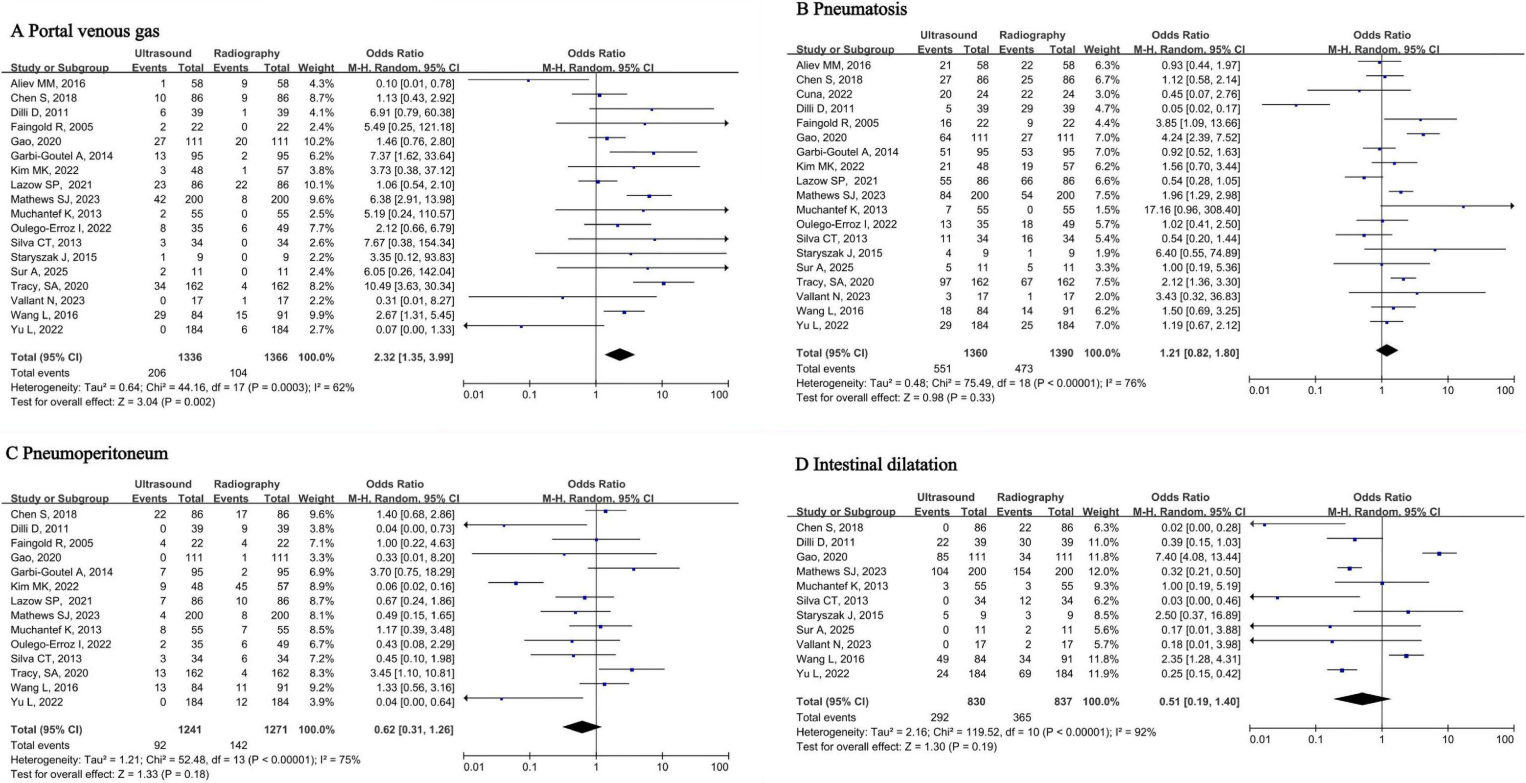
Forest plot comparing the imaging features of AUS vs. AXR for diagnosing NEC. **(A)** Portal venous gas; **(B)** Pneumatosis; **(C)** Pneumoperitoneum; **(D)** Intestinal dilatation.

AUS detected portal venous gas more frequently than AXR [OR: 2.32; 95% CI (1.35–3.99)]. AUS also showed a higher detection frequency for pneumatosis intestinalis as well, though this frequency did not reach statistical significance [OR: 1.21; 95% CI (0.82–1.80)] AXR detected pneumoperitoneum [OR: 0.62; 95% CI (0.31–1.26)] and intestinal dilatation [OR: 0.51; 95% CI (0.19–1.40)] more frequently than AUS, though neither frequency was statistically significant (Figure 2). For other signs, including bowel wall thinning, bowel wall thickening, decreased intestinal peristalsis, abdominal fluid accumulation, and the absence of or decrease in bowel perfusion, descriptive statistics were used to summarize the data instead of conducting a pooled analysis owing to the extremely low event rates in the AXR group (Supplementary Table S2). Considering that these signs are inherently detectable by ultrasonography but not by radiography, zero detection of the signs by AXR reflects a physical/technical constraint rather than the relatively diagnostic superiority of AUS. These findings consistently demonstrated higher detection rates with AUS compared to AXR, except in cases involving gasless abdomen, where AXR showed higher detection rates than AUS.

### 3.3 Publication bias

**Figure 3:**
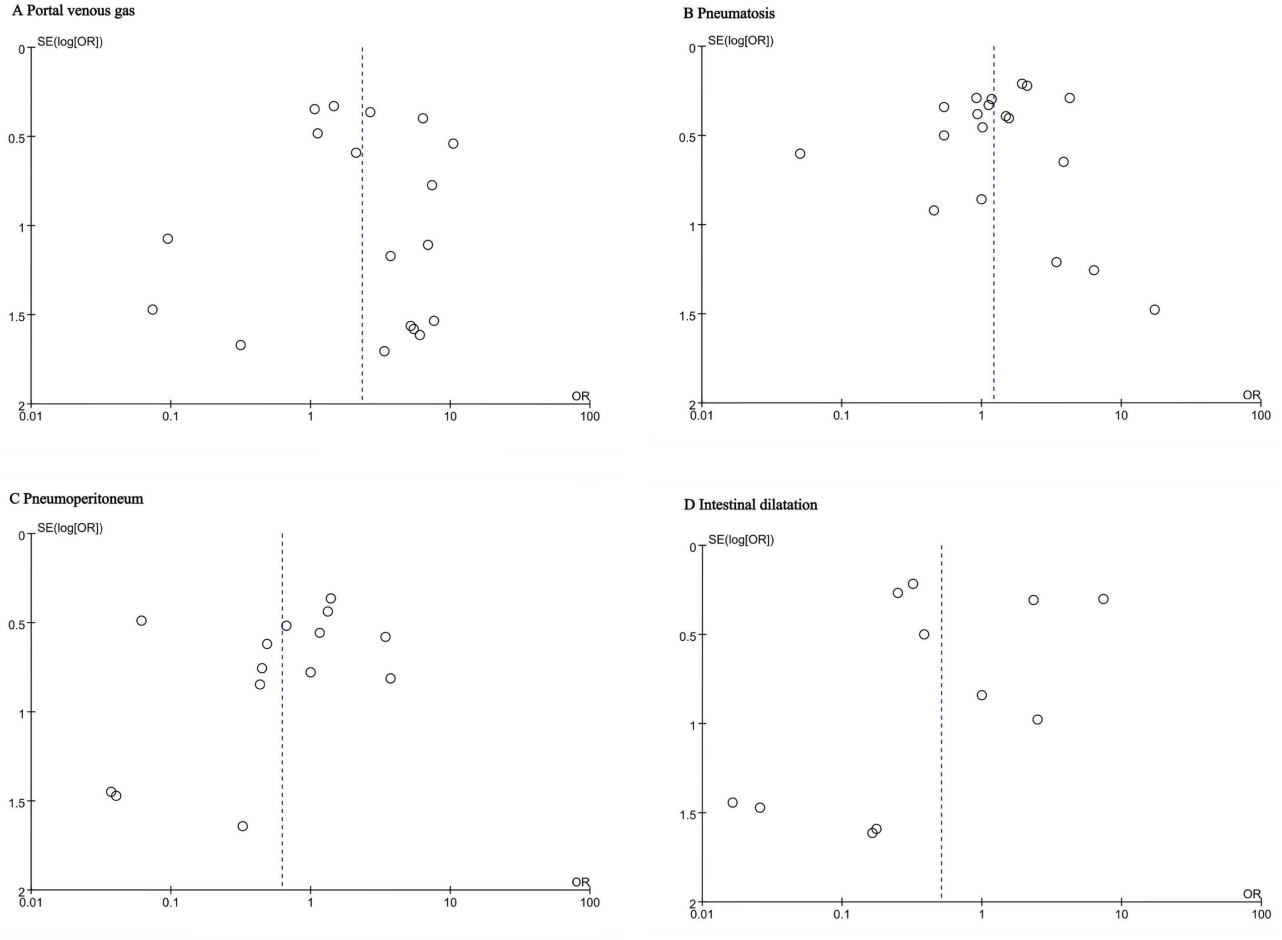
Funnel plot comparing the imaging features of AUS vs. AXR for diagnosing NEC. **(A)** Portal venous gas; **(B)** Pneumatosis; **(C)** Pneumoperitoneum; **(D)** Intestinal dilatation.

Funnel plots were used to assess publication bias (Figure 3). The *P*-values of Egger's test for portal venous gas, pneumatosis, pneumoperitoneum, and intestinal dilatation were 0.391, 0.351, 0.706, and 0.912, respectively. Due to the limited number of studies, the results from Egger's test should be interpreted cautiously, and publication bias cannot be confidently excluded.

### 3.4 GRADE assessment

The certainty of evidence was assessed using the GRADE approach (Table 3). The certainty was rated as very low for portal venous gas. The initial certainty was upgraded by one level because of a large effect size but downgraded by one level owing to substantial heterogeneity and by one level for risk of bias (most studies used paired designs but only reported independent rates). For pneumatosis intestinalis, pneumoperitoneum, and intestinal dilatation, the certainty was rated as very low, downgraded for high heterogeneity and imprecision (95% CI crosses the null).

**Table 3:** The GRADE assessment.

Patient or population: Patients with confirmed or suspected necrotizing enterocolitis						
Settings: Neonatology						
Intervention: Ultrasound						
Comparison: Abdominal Radiography						
Outcomes	Illustrative comparative risks^a^ (95% CI)		Relative effect (95% CI)	No of Participants (studies)	Quality of the evidence (GRADE)	Comments
	Assumed risk	Corresponding risk				
	Radiography	Ultrasound				
Portal venous gas	76 per 1,000	160 per 1,000 (100 to 247)	OR 2.31 (1.35 to 3.99)	2,702 (18 studies)	⊕⊝⊝⊝ very low^b^^,^^c^^,^^d^	
Pneumatosis	340 per 1,000	384 per 1,000 (297 to 481)	OR 1.21 (0.82 to 1.80)	2,750 (19 studies)	⊕⊝⊝⊝ very low^e^^,^^d^^,^^f^	
Pneumoperitoneum	112 per 1,000	72 per 1,000 (38 to 137)	OR 0.62 (0.31 to 1.26)	2,512 (15 studies)	⊕⊝⊝⊝ very low^e^^,^^e^^,^^f^	
Intestinal dilatation	317 per 1,000	191 per 1,000 (81 to 393)	OR 0.51 (0.19 to 1.40)	1,667 (11 studies)	⊕⊝⊝⊝ very low^e^^,^^g^^,^^f^^,^^h^	

a The basis for the assumed risk (e.g., the median control group risk across studies) is provided in footnotes. The corresponding risk (and its 95% confidence interval) is based on the assumed risk in the comparison group and the relative effect of the intervention (and its 95% CI).

CI: Confidence interval; OR: Odds ratio.

GRADE Working Group grades of evidence.

High quality: Further research is very unlikely to change our confidence in the estimate of effect.

Moderate quality: Further research is likely to have an important impact on our confidence in the estimate of effect and may change the estimate.

Low quality: Further research is very likely to have an important impact on our confidence in the estimate of effect and is likely to change the estimate.

Very low quality: We are very uncertain about the estimate.

b Moderate heterogeneity.

c RR > 2.0.

d Most studies used paired designs but only reported independent rates; not analyzed using paired methods. 95% confidence interval (CI) may be too narrow.

e High heterogeneity.

f 95% CI crosses the null value (OR=1.0).

g Paired data not analyzed using paired methods may narrow 95% CI.

h RR = 0.74.

### 3.5 Sensitivity analysis

Leave-one-out sensitivity analysis showed that the pooled estimates for pneumatosis intestinalis and intestinal dilatation were influenced by single studies. For pneumatosis intestinalis, excluding the study by Dilli (15) changed the pooled OR from 1.21 [95% CI (0.82–1.80)] to 1.41 [95% CI (1.03–1.93)]. For intestinal dilatation, excluding the study by Gao (17) changed the pooled OR from 0.51 [95% CI (0.19–1.40)] to 0.40 [95% CI (0.18–0.91)]. Exclusion of studies with an NOS score of 7 did not materially alter any estimate. Detailed variance components are in Supplementary Table S3. In the variance-inflation sensitivity analysis restricted to paired-design studies (*n* = X), applying ICC = 0.3 and 0.5 yielded pooled ORs that remained directionally consistent with the primary analysis for all four imaging signs (Table 4). Notably, only portal venous gas under the most conservative ICC = 0.5 correction showed a CI marginally crossing 1.0 [2.07 (0.98–4.36)], indicating that this finding is the most sensitive to paired-data correction.

**Table 4:** Sensitivity analysis of variance inflation under assumed ICC values for paired-design studies.

Imaging sign	Unadjusted OR (95% CI)	ICC=0.3 OR (95% CI)	ICC=0.5 OR (95% CI)
Portal venous gas	2.32 (1.35–3.99)	2.07 (1.10–3.88)	2.07 (0.98–4.36)
Pneumatosis	1.21 (0.82–1.80)	1.16 (0.70–1.92)	1.16 (0.64–2.11)
Pneumoperitoneum	0.62 (0.31–1.26)	0.61 (0.23–1.59)	0.61 (0.20–1.89)
Intestinal dilatation	0.51 (0.19–1.40)	0.51 (0.15–1.68)	0.51 (0.12–2.09)

ICC, intraclass correlation coefficient; OR, odds ratio; CI, confidence interval.

### 3.6 Subgroup analyses

Subgroup analyses were performed to explore potential sources of heterogeneity according to gestational age, NEC definition, and surgical verification status (Supplementary Table S4). Interaction *P*-values are presented in Supplementary Table S5. For portal venous gas and pneumatosis intestinalis, AUS descriptively showed higher detection rates in preterm or <32 weeks subgroups and in those using (modified) Bell's criteria; however, none of the interaction tests reached statistical significance (all *P* > 0.05). For intestinal dilatation, AXR descriptively showed higher detection rates in surgically verified studies (OR: 0.31, 95% CI 0.20–0.42), but the interaction test did not reach significance (*P* = 0.41). For pneumoperitoneum, a significant subgroup interaction was observed by surgical verification status (*P* = 0.0003, *I*^2^ = 92.3%); this finding should be considered exploratory. For all other subgroup comparisons, interaction *P*-values were >0.05 with *I*^2^ = 0%, indicating no statistically significant subgroup differences.

## 4 Discussion

AXR has long been used as a primary imaging modality for evaluating NEC, assessing its severity, and monitoring its progression (6, 30). However, the present results indicate that while AXR may detect pneumoperitoneum, gasless abdomen, and intestinal dilatation more frequently than AUS, it detects pneumatosis intestinalis, bowel wall thickening, abdominal fluid accumulation, decreased intestinal peristalsis, bowel wall thinning, absent or decreased bowel perfusion, hyperemic bowel, and increased wall echogenicity less frequently than AUS. These findings highlight the notable limitations of AXR, which often make the early recognition of NEC features challenging (31).

The increasing implementation of AUS in neonatal care is largely attributed to its favorable safety profile and convenience. Furthermore, several studies have indicated that AUS can detect early pathological features, including abnormal perfusion, intramural gas, and portal venous gas (9, 26, 32, 33), more readily than conventional radiography potentially enabling earlier recognition and therapeutic intervention (9, 31). This rapid detection capability is critical for ensuring timely surgical decision-making and improving clinical outcomes. The presence of pneumoperitoneum on plain abdominal radiography may be indicative of advanced perforated NEC, whereas AUS can detect abnormal signs, such as complex ascites and bowel wall thinning or thickening before free air is visible on AXR. These signs may serve as early indicators of intestinal perforation and are important factors to consider when adjusting treatment strategies. Furthermore, when clinical deterioration occurs during the course of NEC, the detection of intestinal changes based on AUS may be a critical determinant in guiding surgical decision-making and may improve clinical outcomes in infants with NEC (8, 15, 20, 21, 28). Additionally, AUS can track the evolution of bowel wall changes at the bedside—namely thickening and hyperemia, thinning and ischemia, and necrosis and perforation—serving as a convenient and safe technique for perioperative intestinal status monitoring (9).

The subgroup analyses revealed that descriptively, AUS showed higher detection rates of pneumatosis intestinalis in very preterm infants (<32 weeks) and in studies using standardized (modified) Bell's criteria; however, the interaction tests for both subgroup comparisons yielded non-significant results (*P* = 0.85 and *P* = 0.49, respectively). Therefore, these findings should be considered exploratory rather than confirmatory. Conversely, AXR showed higher detection rates than AUS for intestinal dilatation in studies with surgical verification; however, this interaction test was also non-significant (*P* = 0.41), suggesting that surgical verification status did not modify the between-modality difference.

Previous studies have linked specific AUS findings, namely complex ascites, bowel wall thinning with loss of perfusion, and absent bowel perfusion, to surgical NEC (12, 14). Our meta-analysis advances this question by systematically quantifying the detection rates of these signs. In our subgroup analysis, AXR detected intestinal dilatation more frequently than AUS in surgically confirmed cases; however, this finding emerged from an exploratory analysis that included a limited number of studies and should be interpreted as hypothesis-generating rather than confirmatory. Confounding by disease severity represents a plausible alternative explanation. Our subgroup analysis also aligns with the findings of Faingold et al. (16) (absent bowel perfusion as a surgical marker) and Kim et al. (20) (sonographic perforation without pneumoperitoneum), indicating that no single sign dictates surgery. AXR-detected dilatation may raise suspicion, whereas AUS findings like absent perfusion or sonographic perforation add weight toward exploration when signs are equivocal. Nonetheless, current data are insufficient to define a formal threshold. Future prospective studies are needed to validate the predictive value of specific AUS findings for surgical outcomes.

For pneumoperitoneum, a significant subgroup interaction was observed by surgical verification status (*P* = 0.0003), although this finding should be considered exploratory given the limited number of studies and high interaction I² (92.3%). One possible explanation is that surgically verified cases represent a more severely ill population with a higher prevalence of free intraperitoneal air, which may be captured differently across imaging modalities in this context. In non-surgically verified cases, the distinction between free air and overlapping bowel gas loops may pose greater interpretive ambiguity for both AUS and AXR. Given the exploratory nature of this analysis and the multiple subgroup comparisons performed, this finding requires confirmation in future prospective studies with standardized verification protocols.

Despite these benefits, the lack of consensus guidelines and technical limitations have created barriers to the routine use of AUS, and many clinicians remain reluctant to base management decisions solely on AUS findings. The present study addresses this gap by synthesizing evidence supporting the use of AUS as an effective imaging modality for identifying portal venous gas, bowel wall abnormalities, and ascites. In the sensitivity analysis, exclusion of Dilli et al. (15) shifted the pooled OR for pneumatosis intestinalis from non-significant to significant; similarly, exclusion of Gao et al. (17) changed the OR for intestinal dilatation from non-significant to significant. A notable common methodological feature of these studies was that they included suspected NEC cases in addition to confirmed cases, whereas most other included studies enrolled only confirmed NEC cases. The inclusion of milder, suspected cases may have attenuated the between-modality differences observed in the confirmed NEC population, which may partly explain their disproportionate influence on the pooled estimates. These findings suggest that our results are more robust in the confirmed NEC population and underscore the need for caution when interpreting pooled estimates across studies using heterogeneous NEC definitions. However, GRADE assessment indicated very low certainty of evidence for all outcomes. The higher detection rates of AUS may broaden the window for surgical decision-making, which is crucial for improving patient outcomes, controlling disease progression, and reducing long-term complications. Nevertheless, the evidence was derived solely from patients with suspected or confirmed NEC, and it was not possible to evaluate the diagnostic sensitivity or specificity, or to establish a diagnostic model, which are issues that should be explored further in future research.

### 4.1 Strengths and limitations

Beyond its systematic and comprehensive multi-database search strategy, this study has several methodological strengths. These include formal classification of included studies by paired vs. unpaired designs with variance-inflation sensitivity analyses using assumed ICC values, subgroup analyses with formal interaction testing across key clinical covariates, and GRADE assessment with conservative downgrading for all outcomes. These features distinguish this work from prior reviews in neonatal NEC imaging.

This meta-analysis had some limitations that should be acknowledged. First, the evidence base was constrained by the limited number of included studies, only one of which was an RCT. Second, the search was restricted to English-language databases, introducing the potential for language bias and leading to the omission of potentially relevant non-English studies. Third, the present study only compared the ability of the two imaging modalities to detect abnormal abdominal signs in patients with NEC, and it did not compare the diagnostic performance of AUS and AXR; therefore, more rigorous diagnostic studies will be required to address this gap. Fourth, the diagnostic criteria for NEC were not uniformly applied across studies, with some including suspected alongside confirmed cases—a feature that may have introduced clinical heterogeneity and, as our sensitivity analysis suggested, exerted a disproportionate influence on the pooled estimates. Fifth, substantial heterogeneity was observed for pneumatosis intestinalis (*I*^2^ = 76%), pneumoperitoneum (*I*^2^ = 75%), and intestinal dilatation (*I*^2^ = 92%). Although subgroup analyses helped explain some of this variance, the leave-one-out sensitivity analysis indicated that the results for pneumatosis intestinalis and intestinal dilatation were influenced by single studies. These findings should therefore be interpreted with caution, and future studies are needed to confirm their robustness. Sixth, individual-level paired data were unavailable, and we categorized study designs and performed variance-inflated sensitivity analyses with ICC values of 0.3–0.5 (Tables 4) to assess the potential impact of non-independence. The overall signal remained consistent across these corrections, although the result for portal venous gas was sensitive to the most extreme correction (ICC = 0.5). These related findings should therefore be interpreted with caution. Finally, the lack of standardized scoring systems for AUS and AXR findings, as well as the absence of uniform severity grading criteria across studies, precluded the development of a predictive diagnostic model for NEC.

## 5 Conclusion

Although AUS and AXR exhibit complementary strengths, AXR continues to serve as the primary imaging modality for NEC in clinical practice. Nevertheless, the present findings confirm that AUS is a powerful tool that can facilitate the early recognition of NEC features, particularly owing to its higher detection rates of portal venous gas and other early signs, without requiring ionizing radiation exposure. Subgroup analyses revealed that AUS descriptively detected pneumatosis intestinalis at higher rates in very preterm infants (<32 weeks) and studies that used Bell's criteria; however, the interaction tests did not reach statistical significance. AXR descriptively detected intestinal dilatation at higher rates in surgically verified cases, but this interaction test was also non-significant. These subgroup findings should be considered exploratory. While signs such as AXR-detected dilatation or AUS-detected absent perfusion may inform surgical decisions, current data remain insufficient to define a precise threshold or scoring system integrating both modalities.

### 5.1 Protocol amendments

Two amendments have been made to the registered protocol (original registration date: 25 September 2025): The title has been changed from “Advantages of Ultrasound compared to Abdominal Radiography for the Diagnosis and Prognostication of Necrotizing Enterocolitis: A Systematic Review and Meta-Analysis” to “Comparison of Detection Frequencies Between Ultrasonography and Abdominal Radiography for Abnormal Signs in Necrotizing Enterocolitis: A Systematic Review and Meta-Analysis” to more accurately reflect the primary objective of the review (focusing on detection of abnormal signs rather than diagnosis and prognostication). Literature search date range extension: The original inclusion cut-off date (25 July 2025) has been extended to 1 April 2026 to incorporate more recently published studies. No other changes have been made to the protocol.

## Data Availability

The original contributions presented in the study are included in the article/Supplementary Material, further inquiries can be directed to the corresponding author.
