# Supplementary material for: Comparison of detection frequencies between ultrasonography and abdominal radiography for abnormal signs in necrotizing enterocolitis: a systematic review and meta-analysis
Source: Front Pediatr. 2026 Jul 29;14:1889902. doi: 10.3389/fped.2026.1889902 (PMC13461621; doi:10.3389/fped.2026.1889902)
Supplement: Supplementary file 1 [file Table2.docx]

Supplementary Table 2 Comparison of AUS and AXR for detecting signs of necrotizing enterocolitis: differences in detection rates.

A Bowel wall thinning

| Study  (first author and year) | AUS  (n/N) | AXR  (n/N) | differences in detection rates  (AUS - AXR) |
| --- | --- | --- | --- |
| Sur A, 2025 | 2/11 | 0/11 | +18.18% |
| Mathews SJ, 2023 | 30/200 | 0/200 | +15% |
| Lazow SP, 2021 | 11/86 | 0/86 | +12.79% |
| Tracy, SA, 2020 | 17/162 | 0/162 | +10.49% |
| Chen S, 2018 | 13/86 | 0/86 | +15.11% |
| Aliev MM, 2016 | 9/58 | 0/58 | +15.51% |
| Silva CT, 2013 | 10/34 | 0/34 | +29.41% |
| Muchantef K, 2013 | 11/55 | 0/55 | +20% |
| Dilli D, 2011 | 2/39 | 0/39 | +5.12% |
| Faingold R, 2005 | 8/22 | 0/22 | +36.36% |

B Bowel wall thickening

| Study  (first author and year) | AUS  (n/N) | AXR  (n/N) | differences in detection rates  (AUS - AXR) |
| --- | --- | --- | --- |
| Sur A, 2025 | 1/11 | 1/11 | 0 |
| Mathews SJ, 2023 | 2/200 | 0/200 | +1% |
| Kim MK, 2022 | 45/48 | 0/57 | +93.75% |
| Yu L, 2022 | 29/184 | 60/184 | -16.84% |
| Lazow SP, 2021 | 23/86 | 0/86 | +26.74% |
| Tracy, SA, 2020 | 34/162 | 17/162 | +10.49% |
| Chen S, 2018 | 20/86 | 0/86 | +23.25% |
| Aliev MM, 2016 | 27/58 | 0/58 | +46.55% |
| Wang L, 2016 | 24/84 | 35/91 | -9.89% |
| Kamali K, 2015 | 3/34 | 0/34 | +8.82% |
| Garbi-Goutel A, 2014 | 31/95 | 0/95 | +32.63% |
| Silva CT, 2013 | 11/34 | 0/34 | +32.35% |
| Muchantef K, 2013 | 30/55 | 0/55 | +54.54% |
| Dilli D, 2011 | 11/39 | 0/39 | +28.2% |
| Faingold R, 2005 | 22/22 | 0/22 | +100% |

C Decreased intestinal peristalsis

| Study  (first author and year) | AUS  (n/N) | AXR  (n/N) | differences in detection rates  (AUS - AXR) |
| --- | --- | --- | --- |
| Sur A, 2025 | 11/11 | 0/11 | +100% |
| Vallant N, 2023 | 4/17 | 0/17 | +23.52% |
| Yu L, 2022 | 27/184 | 0/184 | +14.67% |
| Gao, 2020 | 85/111 | 0/111 | +76.57% |
| Tracy, SA, 2020 | 49/162 | 0/162 | +30.24% |
| Chen S, 2018 | 17/86 | 0/86 | +19.76% |
| Aliev MM, 2016 | 20/58 | 0/58 | +34.48% |
| Wang L, 2016 | 51/84 | 0/91 | +60.71% |
| Staryszak J, 2015 | 4/9 | 0/9 | +44.44% |
| Silva CT, 2013 | 15/34 | 0/34 | +44.11% |
| Muchantef K, 2013 | 21/55 | 0/55 | +38.18% |
| Faingold R, 2005 | 13/22 | 0/22 | +59.09% |

D Abdominal fluid

| Study  (first author and year) | AUS  (n/N) | AXR  (n/N) | differences in detection rates  (AUS - AXR) |
| --- | --- | --- | --- |
| Sur A, 2025 | 5/11 | 1/11 | +36.36% |
| Vallant N, 2023 | 8/17 | 0/17 | +47.05% |
| Oulego-Erroz I, 2022 | 2/35 | 0/49 | +5.71% |
| Mathews SJ, 2023 | 26/200 | 0/200 | +13% |
| Kim MK, 2022 | 48/48 | 0/57 | +100% |
| Lazow SP, 2021 | 21/86 | 0/86 | +24.41% |
| Gao, 2020 | 11/111 | 0/111 | +9.9% |
| Tracy, SA, 2020 | 35/162 | 0/162 | +21.6% |
| Chen S, 2018 | 42/86 | 0/86 | +48.83% |
| Aliev MM, 2016 | 31/58 | 7/58 | +41.37% |
| Wang L, 2016 | 56/84 | 0/91 | +66.66 |
| Staryszak J, 2015 | 6/9 | 0/9 | +66.66% |
| Kamali K, 2015 | 16/34 | 0/34 | +47.05% |
| Garbi-Goutel A, 2014 | 23/95 | 0/95 | +24.21% |
| Dilli D, 2011 | 22/39 | 0/39 | +56.41% |
| Faingold R, 2005 | 9/22 | 0/22 | +40.9% |

E Absent or decreased bowel perfusion

| Study  (first author and year) | AUS  (n/N) | AXR  (n/N) | differences in detection rates  (AUS - AXR) |
| --- | --- | --- | --- |
| Sur A, 2025 | 3/11 | 0/11 | +27.27% |
| Kim MK, 2022 | 7/48 | 0/57 | +14.58% |
| Lazow SP, 2021 | 14/86 | 0/86 | +16.27% |
| Tracy, SA, 2020 | 28/162 | 0/162 | +17.28% |
| Aliev MM, 2016 | 10/58 | 0/58 | +17.24% |
| Silva CT, 2013 | 3/34 | 0/34 | +8.82% |
| Muchantef K, 2013 | 12/55 | 0/55 | +21.81% |
| Faingold R, 2005 | 12/22 | 0/22 | +54.54% |

F Hyperemic bowel

| Study  (first author and year) | AUS  (n/N) | AXR  (n/N) | differences in detection rates  (AUS - AXR) |
| --- | --- | --- | --- |
| Sur A, 2025 | 3/11 | 0/11 | +27.27% |
| Lazow SP, 2021 | 12/86 | 0/86 | +13.95% |
| Aliev MM, 2016 | 19/58 | 0/58 | +32.75% |
| Silva CT, 2013 | 9/34 | 0/34 | +26.47% |
| Muchantef K, 2013 | 26/55 | 0/55 | +47.27% |

G Increased wall echogenicity

| Study  (first author and year) | AUS  (n/N) | AXR  (n/N) | differences in detection rates  (AUS - AXR) |
| --- | --- | --- | --- |
| Lazow SP, 2021 | 14/86 | 0/86 | +16.27% |
| Kamali K, 2015 | 1/34 | 0/34 | +2.94% |
| Silva CT, 2013 | 23/34 | 0/34 | +67.64% |
| Muchantef K, 2013 | 30/55 | 0/55 | +54.54% |

H Gasless abdomen

| Study  (first author and year) | AUS  (n/N) | AXR  (n/N) | differences in detection rates  (AUS - AXR) |
| --- | --- | --- | --- |
| Sur A, 2025 | 0/11 | 3/11 | -27.27% |
| Vallant N, 2023 | 0/17 | 13/17 | -76.47% |
| Kim MK, 2022 | 0/48 | 10/57 | -17.54% |
| Gao, 2020 | 14/111 | 11/111 | +27.27% |
| Chen S, 2018 | 0/86 | 25/86 | -29.06% |
| Wang L, 2016 | 34/84 | 23/91 | +15.19% |
| Staryszak J, 2015 | 0/9 | 2/9 | -22.22% |
| Muchantef K, 2013 | 0/55 | 21/55 | -38.18% |
