# Supplementary material for: Comparison of detection frequencies between ultrasonography and abdominal radiography for abnormal signs in necrotizing enterocolitis: a systematic review and meta-analysis
Source: Front Pediatr. 2026 Jul 29;14:1889902. doi: 10.3389/fped.2026.1889902 (PMC13461621; doi:10.3389/fped.2026.1889902)
Supplement: Supplementary file 2 [file Table3.docx]

Supplementary Table 3 Sensitivity analyses

|  | Analysis | OR (95% CI) |
| --- | --- | --- |
| Portal venous gas | Original | 2.32 [1.35, 3.99] |
|  | After excluding Aliev MM, 2016 | 2.66 [1.60, 4.41] |
|  | After excluding Chen S, 2018 | 2.48 [1.39, 4.43] |
|  | After excluding Dilli D, 2011 | 2.21 [1.27, 3.87] |
|  | After excluding Faingold R, 2005 | 2.27 [1.30, 3.95] |
|  | After excluding Gao, 2020 | 2.43 [1.33, 4.45] |
|  | After excluding Garbi-Goutel A, 2014 | 2.15 [1.23, 3.76] |
|  | After excluding Kim MK, 2022 | 2.28 [1.30, 3.99] |
|  | After excluding Lazow SP, 2021 | 2.53 [1.42, 4.50] |
|  | After excluding Mathews SJ, 2023 | 2.08 [1.20, 3.61] |
|  | After excluding Muchantef K, 2013 | 2.27 [1.30, 3.96] |
|  | After excluding Oulego-Erroz I, 2022 | 2.33 [1.30, 4.19] |
|  | After excluding Silva CT, 2013 | 2.25 [1.29, 3.91] |
|  | After excluding Staryszak J, 2015 | 2.30 [1.32, 4.01] |
|  | After excluding Sur A, 2025 | 2.27 [1.30, 3.95] |
|  | After excluding Tracy, SA, 2020 | 2.03 [1.20, 3.44] |
|  | After excluding Vallant N, 2023 | 2.43 [1.40, 4.20] |
|  | After excluding Wang L, 2016 | 2.28 [1.24, 4.19] |
|  | After excluding Yu L, 2022 | 2.55 [1.51, 4.30] |
|  | After excluding studies with an NOS score of 7:  Yu L, 2022  Lazow SP, 2021  Garbi-Goutel A, 2014  Silva CT, 2013 | 2.26 [1.35, 3.78] |
|  | After excluding unpaired studies:  Faingold R, 2005  Oulego-Erroz I, 2022 | 2.07 [1.22, 3.50] |
| Pneumatosis | Original | 1.21 [0.82, 1.80] |
|  | After excluding Aliev MM, 2016 | 1.24 [0.82, 1.87] |
|  | After excluding Chen S, 2018 | 1.22 [0.80, 1.86] |
|  | After excluding Cuna, 2022 | 1.25 [0.84, 1.86] |
|  | After excluding Dilli D, 2011 | 1.41 [1.03, 1.93] |
|  | After excluding Faingold R, 2005 | 1.15 [0.77, 1.72] |
|  | After excluding Gao, 2020 | 1.10 [0.76, 1.61] |
|  | After excluding Garbi-Goutel A, 2014 | 1.24 [0.82, 1.88] |
|  | After excluding Kim MK, 2022 | 1.20 [0.79, 1.81] |
|  | After excluding Lazow SP, 2021 | 1.29 [0.87, 1.91] |
|  | After excluding Mathews SJ, 2023 | 1.17 [0.76, 1.80] |
|  | After excluding Muchantef K, 2013 | 1.17 [0.79, 1.72] |
|  | After excluding Oulego-Erroz I, 2022 | 1.23 [0.81, 1.85] |
|  | After excluding Silva CT, 2013 | 1.27 [0.85, 1.90] |
|  | After excluding Staryszak J, 2015 | 1.18 [0.79, 1.74] |
|  | After excluding Sur A, 2025 | 1.22 [0.82, 1.83] |
|  | After excluding Tracy, SA, 2020 | 1.16 [0.76, 1.77] |
|  | After excluding Vallant N, 2023 | 1.19 [0.80, 1.77] |
|  | After excluding Wang L, 2016 | 1.20 [0.79, 1.81] |
|  | After excluding Yu L, 2022 | 1.22 [0.80, 1.86] |
|  | After excluding studies with an NOS score of 7:  Yu L, 2022  Lazow SP, 2021  Garbi-Goutel A, 2014  Silva CT, 2013 | 1.43 [0.89, 2.29] |
|  | After excluding unpaired studies:  Faingold R, 2005  Oulego-Erroz I, 2022 | 1.16 [0.76, 1.77] |
| Pneumoperitoneum | Original | 0.62 [0.31, 1.26] |
|  | After excluding Chen S, 2018 | 0.56 [0.26, 1.23] |
|  | After excluding Dilli D, 2011 | 0.70 [0.35, 1.40] |
|  | After excluding Faingold R, 2005 | 0.60 [0.28, 1.26] |
|  | After excluding Gao, 2020 | 0.63 [0.31, 1.31] |
|  | After excluding Garbi-Goutel A, 2014 | 0.55 [0.27, 1.12] |
|  | After excluding Kim MK, 2022 | 0.88 [0.52, 1.49] |
|  | After excluding Lazow SP, 2021 | 0.61 [0.28, 1.32] |
|  | After excluding Mathews SJ, 2023 | 0.63 [0.29, 1.34] |
|  | After excluding Muchantef K, 2013 | 0.58 [0.27, 1.25] |
|  | After excluding Oulego-Erroz I, 2022 | 0.63 [0.30, 1.33] |
|  | After excluding Silva CT, 2013 | 0.63 [0.30, 1.34] |
|  | After excluding Tracy, SA, 2020 | 0.54 [0.26, 1.09] |
|  | After excluding Wang L, 2016 | 0.57 [0.26, 1.23] |
|  | After excluding Yu L, 2022 | 0.70 [0.35, 1.40] |
|  | After excluding studies with an NOS score of 7:  Yu L, 2022  Lazow SP, 2021  Garbi-Goutel A, 2014  Silva CT, 2013 | 0.62 [0.26, 1.46] |
|  | After excluding unpaired studies:  Faingold R, 2005  Oulego-Erroz I, 2022 | 0.61 [0.27, 1.34] |
| Intestinal dilatation | Original (no unpaired study) | 0.51 [0.19, 1.40] |
|  | After excluding Chen S, 2018 | 0.65 [0.23, 1.80] |
|  | After excluding Dilli D, 2011 | 0.52 [0.17, 1.57] |
|  | After excluding Gao, 2020 | 0.40 [0.18, 0.91] |
|  | After excluding Mathews SJ, 2023 | 0.52 [0.16, 1.69] |
|  | After excluding Muchantef K, 2013 | 0.47 [0.16, 1.39] |
|  | After excluding Silva CT, 2013 | 0.62 [0.22, 1.75] |
|  | After excluding Staryszak J, 2015 | 0.44 [0.15, 1.27] |
|  | After excluding Sur A, 2025 | 0.55 [0.19, 1.55] |
|  | After excluding Vallant N, 2023 | 0.54 [0.19, 1.55] |
|  | After excluding Wang L, 2016 | 0.41 [0.14, 1.25] |
|  | After excluding Yu L, 2022 | 0.55 [0.18, 1.70] |
|  | After excluding studies with an NOS score of 7:  Yu L, 2022  Silva CT, 2013 | 0.70 [0.22, 2.21] |

OR, odds ratio; CI, confidence interval.
