# Supplementary material for: Comparison of detection frequencies between ultrasonography and abdominal radiography for abnormal signs in necrotizing enterocolitis: a systematic review and meta-analysis
Source: Front Pediatr. 2026 Jul 29;14:1889902. doi: 10.3389/fped.2026.1889902 (PMC13461621; doi:10.3389/fped.2026.1889902)
Supplement: Supplementary file 3 [file Table4.docx]

Supplementary Table 4 Subgroup analyses based on gestational age (A), necrotizing enterocolitis definition (B), and surgical verification status (C).

A Gestational age

|  | Included studies | Odds ratios | 95% confidence intervals |
| --- | --- | --- | --- |
| portal venous gas |  |  |  |
| neonates | Lazow SP, 2021 | 2.29 | 0.93~5.61 |
|  | Mathews SJ, 2023 |  |  |
|  | Muchantef K, 2013 |  |  |
|  | Silva CT, 2013 |  |  |
|  | Staryszak J, 2015 |  |  |
|  | Wang L, 2016 |  |  |
|  | Yu L, 2022 |  |  |
| preterm infant | Aliev MM, 2016 | 2.33 | 1.10~4.94 |
|  | Chen S, 2018 |  |  |
|  | Dilli D, 2011 |  |  |
|  | Faingold R, 2005 |  |  |
|  | Gao, 2020 |  |  |
|  | Garbi-Goutel A, 2014 |  |  |
|  | Kim MK, 2022 |  |  |
|  | Oulego-Erroz I, 2022 |  |  |
|  | Sur A, 2025 |  |  |
|  | Tracy, SA, 2020 |  |  |
|  | Vallant N, 2023 |  |  |
| <32 weeks | Kim MK, 2022 | 4.90 | 1.35~17.78 |
|  | Sur A, 2025 |  |  |
|  | Tracy, SA, 2020 |  |  |
|  | Vallant N, 2023 |  |  |
| pneumatosis |  |  |  |
| neonates | Lazow SP, 2021 | 1.25 | 0.72~2.18 |
|  | Mathews SJ, 2023 |  |  |
|  | Muchantef K, 2013 |  |  |
|  | Silva CT, 2013 |  |  |
|  | Staryszak J, 2015 |  |  |
|  | Wang L, 2016 |  |  |
|  | Yu L, 2022 |  |  |
| preterm infant | Aliev MM, 2016 | 1.16 | 0.66~2.02 |
|  | Chen S, 2018 |  |  |
|  | Cuna, 2022 |  |  |
|  | Dilli D, 2011 |  |  |
|  | Faingold R, 2005 |  |  |
|  | Gao, 2020 |  |  |
|  | Garbi-Goutel A, 2014 |  |  |
|  | Kim MK, 2022 |  |  |
|  | Oulego-Erroz I, 2022 |  |  |
|  | Sur A, 2025 |  |  |
|  | Tracy, SA, 2020 |  |  |
|  | Vallant N, 2023 |  |  |
| <32 weeks | Cuna, 2022 | 1.67 | 1.19~2.35 |
|  | Kim MK, 2022 |  |  |
|  | Sur A, 2025 |  |  |
|  | Tracy, SA, 2020 |  |  |
|  | Vallant N, 2023 |  |  |
| pneumoperitoneum |  |  |  |
| neonates | Lazow SP, 2021 | 0.70 | 0.37~1.32 |
|  | Mathews SJ, 2023 |  |  |
|  | Muchantef K, 2013 |  |  |
|  | Silva CT, 2013 |  |  |
|  | Wang L, 2016 |  |  |
|  | Yu L, 2022 |  |  |
| preterm infant | Chen S, 2018 | 0.61 | 0.18~2.11 |
|  | Dilli D, 2011 |  |  |
|  | Faingold R, 2005 |  |  |
|  | Gao, 2020 |  |  |
|  | Garbi-Goutel A, 2014 |  |  |
|  | Kim MK, 2022 |  |  |
|  | Oulego-Erroz I, 2022 |  |  |
|  | Tracy, SA, 2020 |  |  |
| <32 weeks | Kim MK, 2022 | 0.46 | 0.01~23.93 |
|  | Tracy, SA, 2020 |  |  |
| intestinal dilatation |  |  |  |
| neonates | Mathews SJ, 2023 | 0.58 | 0.21~1.59 |
|  | Muchantef K, 2013 |  |  |
|  | Silva CT, 2013 |  |  |
|  | Staryszak J, 2015 |  |  |
|  | Wang L, 2016 |  |  |
|  | Yu L, 2022 |  |  |
| preterm infant | Chen S, 2018 | 0.34 | 0.03~3.57 |
|  | Dilli D, 2011 |  |  |
|  | Gao, 2020 |  |  |
|  | Sur A, 2025 |  |  |
|  | Vallant N, 2023 |  |  |
| <32 weeks | Sur A, 2025 | 0.17 | 0.02~1.57 |
|  | Vallant N, 2023 |  |  |

B Definition of necrotizing enterocolitis

|  | Included studies | Odds ratios | 95% confidence intervals |
| --- | --- | --- | --- |
| portal venous gas |  |  |  |
| (Modified) Bell’s criteria | Aliev MM, 2016 | 2.22 | 0.98~5.01 |
|  | Faingold R, 2005 |  |  |
|  | Gao, 2020 |  |  |
|  | Garbi-Goutel A, 2014 |  |  |
|  | Mathews SJ, 2023 |  |  |
|  | Silva CT, 2013 |  |  |
|  | Staryszak J, 2015 |  |  |
|  | Sur A, 2025 |  |  |
|  | Wang L, 2016 |  |  |
|  | Yu L, 2022 |  |  |
| Necrotizing enterocolitis definition by Walsh and Kliegman | Chen S, 2018 | 2.14 | 0.38~11.97 |
|  | Dilli D, 2011 |  |  |
| No clear definition | Kim MK, 2022 | 2.36 | 0.48~11.58 |
|  | Lazow SP, 2021 |  |  |
|  | Tracy, SA, 2020 |  |  |
|  | Vallant N, 2023 |  |  |
| pneumatosis |  |  |  |
| (Modified) Bell’s criteria | Aliev MM, 2016 | 1.53 | 1.00~2.34 |
|  | Faingold R, 2005 |  |  |
|  | Gao, 2020 |  |  |
|  | Garbi-Goutel A, 2014 |  |  |
|  | Mathews SJ, 2023 |  |  |
|  | Silva CT, 2013 |  |  |
|  | Staryszak J, 2015 |  |  |
|  | Sur A, 2025 |  |  |
|  | Wang L, 2016 |  |  |
|  | Yu L, 2022 |  |  |
| Necrotizing enterocolitis definition by Walsh and Kliegman | Chen S, 2018 | 0.25 | 0.01~5.19 |
|  | Dilli D, 2011 |  |  |
| No clear definition | Cuna, 2022 | 1.28 | 0.57~2.89 |
|  | Kim MK, 2022 |  |  |
|  | Lazow SP, 2021 |  |  |
|  | Muchantef K, 2013 |  |  |
|  | Tracy, SA, 2020 |  |  |
|  | Vallant N, 2023 |  |  |
| pneumoperitoneum |  |  |  |
| (Modified) Bell’s criteria | Faingold R, 2005  Gao, 2020 | 0.76 | 0.34~1.71 |
|  | Garbi-Goutel A, 2014 |  |  |
|  | Mathews SJ, 2023 |  |  |
|  | Silva CT, 2013 |  |  |
|  | Wang L, 2016 |  |  |
|  | Yu L, 2022 |  |  |
| Necrotizing enterocolitis definition by Walsh and Kliegman | Chen S, 2018 | 0.31 | 0.01~12.19 |
|  | Dilli D, 2011 |  |  |
| No clear definition | Kim MK, 2022 | 0.63 | 0.11~3.46 |
|  | Lazow SP, 2021 |  |  |
|  | Muchantef K, 2013 |  |  |
|  | Tracy, SA, 2020 |  |  |
| intestinal dilatation |  |  |  |
| (Modified) Bell’s criteria | Gao, 2020 | 0.72 | 0.20~2.60 |
|  | Mathews SJ, 2023 |  |  |
|  | Silva CT, 2013 |  |  |
|  | Staryszak J, 2015 |  |  |
|  | Sur A, 2025 |  |  |
|  | Wang L, 2016 |  |  |
|  | Yu L, 2022 |  |  |
| Necrotizing enterocolitis definition by Walsh and Kliegman | Chen S, 2018 | 0.10 | 0.00~3.55 |
|  | Dilli D, 2011 |  |  |
| No clear definition | Muchantef K, 2013 | 0.18 | 0.01~3.98 |
|  | Vallant N, 2023 |  |  |

C Surgical

|  | Included studies | Odds ratios | 95% confidence intervals |
| --- | --- | --- | --- |
| portal venous gas |  |  |  |
| Surgical Verification Group | Aliev MM, 2016 | 1.78 | 0.6~5.3 |
|  | Dilli D, 2011 |  |  |
|  | Kim MK, 2022 |  |  |
|  | Lazow SP, 2021 |  |  |
|  | Mathews SJ, 2023 |  |  |
|  | Muchantef K, 2013 |  |  |
|  | Tracy, SA, 2020 |  |  |
|  | Vallant N, 2023 |  |  |
|  | Yu L, 2022 |  |  |
| Non-surgical validation group | Chen S, 2018 | 2.06 | 1.41~3.01 |
|  | Faingold R, 2005 |  |  |
|  | Gao, 2020 |  |  |
|  | Garbi-Goutel A, 2014 |  |  |
|  | Oulego-Erroz I, 2022 |  |  |
|  | Silva CT, 2013 |  |  |
|  | Staryszak J, 2015 |  |  |
|  | Sur A, 2025 |  |  |
|  | Wang L, 2016 |  |  |
| pneumatosis |  |  |  |
| Surgical Verification Group | Aliev MM, 2016 | 1.06 | 0.57~1.97 |
|  | Dilli D, 2011 |  |  |
|  | Kim MK, 2022 |  |  |
|  | Lazow SP, 2021 |  |  |
|  | Mathews SJ, 2023 |  |  |
|  | Muchantef K, 2013 |  |  |
|  | Tracy, SA, 2020 |  |  |
|  | Vallant N, 2023 |  |  |
|  | Yu L, 2022 |  |  |
| Non-surgical validation group | Chen S, 2018 | 1.39 | 0.84~2.32 |
|  | Cuna, 2022 |  |  |
|  | Faingold R, 2005 |  |  |
|  | Gao, 2020 |  |  |
|  | Garbi-Goutel A, 2014 |  |  |
|  | Oulego-Erroz I, 2022 |  |  |
|  | Silva CT, 2013 |  |  |
|  | Staryszak J, 2015 |  |  |
|  | Sur A, 2025 |  |  |
|  | Wang L, 2016 |  |  |
| pneumoperitoneum |  |  |  |
| Surgical Verification Group | Dilli D, 2011 | 0.36 | 0.10~1.26 |
|  | Kim MK, 2022 |  |  |
|  | Lazow SP, 2021 |  |  |
|  | Mathews SJ, 2023 |  |  |
|  | Muchantef K, 2013 |  |  |
|  | Tracy, SA, 2020 |  |  |
|  | Yu L, 2022 |  |  |
| Non-surgical validation group | Chen S, 2018 | 1.17 | 0.75~1.82 |
|  | Faingold R, 2005 |  |  |
|  | Gao, 2020 |  |  |
|  | Garbi-Goutel A, 2014 |  |  |
|  | Oulego-Erroz I, 2022 |  |  |
|  | Silva CT, 2013 |  |  |
|  | Wang L, 2016 |  |  |
| intestinal dilatation |  |  |  |
| Surgical Verification Group | Dilli D, 2011 | 0.31 | 0.2~ 0.42 |
|  | Mathews SJ, 2023 |  |  |
|  | Muchantef K, 2013 |  |  |
|  | Vallant N, 2023 |  |  |
|  | Yu L, 2022 |  |  |
| Non-surgical validation group | Chen S, 2018 | 0.63 | 0.12~3.17 |
|  | Gao, 2020 |  |  |
|  | Silva CT, 2013 |  |  |
|  | Staryszak J, 2015 |  |  |
|  | Sur A, 2025 |  |  |
|  | Wang L, 2016 |  |  |
