# Supplementary material for: Comparison of detection frequencies between ultrasonography and abdominal radiography for abnormal signs in necrotizing enterocolitis: a systematic review and meta-analysis
Source: Front Pediatr. 2026 Jul 29;14:1889902. doi: 10.3389/fped.2026.1889902 (PMC13461621; doi:10.3389/fped.2026.1889902)
Supplement: Supplementary file 4 [file Table1.doc]

Supplementary Table 1 Study-level design characteristics (paired vs. unpaired)

| Study | AUS and AXR applied to same cohort? | Design type |
| --- | --- | --- |
| Sur A, 2025 | Yes | Paired |
| Vallant N, 2023 | Yes | Paired |
| Oulego-Erroz I, 2022 | No | Unpaired |
| Mathews SJ, 2023 | Yes | Paired |
| Kim MK, 2022 | Yes | Paired |
| Yu L, 2022 | Yes | Paired |
| Lazow SP, 2021 | Yes | Paired |
| Cuna, 2022 | Yes | Paired |
| Gao, 2020 | Yes | Paired |
| Tracy, SA, 2020 | Yes | Paired |
| Chen S, 2018 | Yes | Paired |
| Aliev MM, 2016 | Yes | Paired |
| Wang L, 2016 | Yes | Paired |
| Staryszak J, 2015 | Yes | Paired |
| Kamali K, 2015 | No | Unpaired |
| Garbi-Goutel A, 2014 | Yes | Paired |
| Silva CT, 2013 | Yes | Paired |
| Muchantef K, 2013 | Yes | Paired |
| Dilli D, 2011 | Yes | Paired |
| Faingold R, 2005 | No | Unpaired |
