# Supplementary material for: Comparison of detection frequencies between ultrasonography and abdominal radiography for abnormal signs in necrotizing enterocolitis: a systematic review and meta-analysis
Source: Front Pediatr. 2026 Jul 29;14:1889902. doi: 10.3389/fped.2026.1889902 (PMC13461621; doi:10.3389/fped.2026.1889902)
Supplement: Supplementary file 5 [file Table5.doc]

Supplementary Table 5 Subgroup analyses for the comparison of AUS and AXR in detecting abnormal findings in patients with NEC (interaction P-values).

| Abnormality | Subgroup Factor | Interaction P-value | I² (%) |
| --- | --- | --- | --- |
| Portal venous gas | Neonates/ preterm infant | 0.98 | 0 |
|  | (Modified) Bell’s criteria/ Necrotizing enterocolitis definition by Walsh and Kliegman/ No clear definition | 1 | 0 |
|  | Surgical Verification Group/ Non-surgical validation group | 0.8 | 0 |
| Pneumatosis | Neonates/ preterm infant | 0.85 | 0 |
|  | (Modified) Bell’s criteria/ Necrotizing enterocolitis definition by Walsh and Kliegman/ No clear definition | 0.49 | 0 |
|  | Surgical Verification Group/ Non-surgical validation group | 0.51 | 0 |
| Pneumoperitoneum | Neonates/ preterm infant | 0.86 | 0 |
|  | (Modified) Bell’s criteria/ Necrotizing enterocolitis definition by Walsh and Kliegman/ No clear definition | 0.88 | 0 |
|  | Surgical Verification Group/ Non-surgical validation group | 0.0003 | 92.3 |
| Intestinal dilatation | Neonates/ preterm infant | 0.68 | 0 |
|  | (Modified) Bell’s criteria/ Necrotizing enterocolitis definition by Walsh and Kliegman/ No clear definition | 0.47 | 0 |
|  | Surgical Verification Group/ Non-surgical validation group | 0.41 | 0 |
